# Giant ovarian cyst masquerading as a massive ascites: a case report

**DOI:** 10.1186/s13104-017-3093-8

**Published:** 2017-12-19

**Authors:** Eugene Vernyuy Yeika, Derrick Tembi Efie, Paul Nkemtendong Tolefac, Joseph Nkeangu Fomengia

**Affiliations:** 1 Saint Elizabeth Catholic General Hospital and Cardiac Centre Shisong, PO Box 08, Kumbo, Cameroon; 2 Clinical Research Education Networking and Consultancy, Douala, Cameroon; 3 Health and Human Development Research Network, Douala, Cameroon; 4 Banso Baptist Hospital, Kumbo, Cameroon

**Keywords:** Giant ovarian cyst, Abdominal distension, Ascites, Case report

## Abstract

**Background:**

Giant ovarian cysts are tumours of the ovary presenting with diameters greater than 10 cm. Giant ovarian cysts have become rare in recent days as they are diagnosed and managed early due to the availability of good imaging modalities. The aim of this case report is to show how a huge cystic ovarian mass can mislead the diagnosis of ascites in a postmenopausal woman. Factors associated with late presentation of giant ovarian cysts in sub-Saharan Africa have also been discussed.

**Case presentation:**

We present the case of a 65-year-old grand multiparous woman who was referred to our centre with a grossly distended abdomen misdiagnosed as a massive ascites. Abdominopelvic ultrasound scan revealed a right giant multiloculated ovarian cyst. She benefited from a cystectomy with an uneventful postoperative stay. Histopathology revealed mucinous cystadenoma.

**Conclusion:**

Large cystic ovarian tumours can present masquerading as massive ascites and misleading diagnosis as in this case report. We report this case to increase the suspicion index of a large ovarian cyst in all women presenting with massive ascites.

## Background

Giant ovarian cysts (GOCs) are rare tumours of the ovary presenting with diameters greater than 10 cm [1, 2]. Ovarian cysts are generally asymptomatic at early stages causing symptoms only after reaching enormous dimensions, and consequently they are often diagnosed late [1, 3, 4]. The clinical symptoms of ovarian cysts are usually progressive abdominal distension, nonspecific diffuse abdominal pain, vaginal bleeding and symptoms related to organs compression such as constipation, early satiety, vomiting and frequent micturition [3, 5–8]. The actual incidence of GOCs in postmenopausal women is unknown since good imaging modalities now lead to early diagnosis and removal before they develop into huge intra-abdominal masses [2–4, 6]. Majority of GOCs are benign and are generally treated by surgical excision either by cystectomy or salpingo-oophorectomy [2–4, 9, 10]. Malignant ovarian cysts (MOC) constitute over 10% of GOCs and are treated by total abdominal hysterectomy with bilateral salpingo-oophorectomy ± omentectomy [5]. Very few cases of GOCs that present masquerading as ascites have been reported. This case is reported to increase the suspicion index of giant intra-abdominal cysts in women presenting with massive ascites. Factors associated with late presentation of GOCs in sub-Saharan Africa (SSA) have also been discussed.

## Case presentation

Herein, we present the case of a 65-year-old black African woman, who was referred to our centre for the management of a massive ascites. She presented with a 5-year history of progressive abdominal distension. She had no abdominal pain, nausea, vomiting or change in bowel habit but complained of increased urinary frequency. She was a grand multipara with parity of 6 and had no relevant past medical history.

**Fig. 1:**
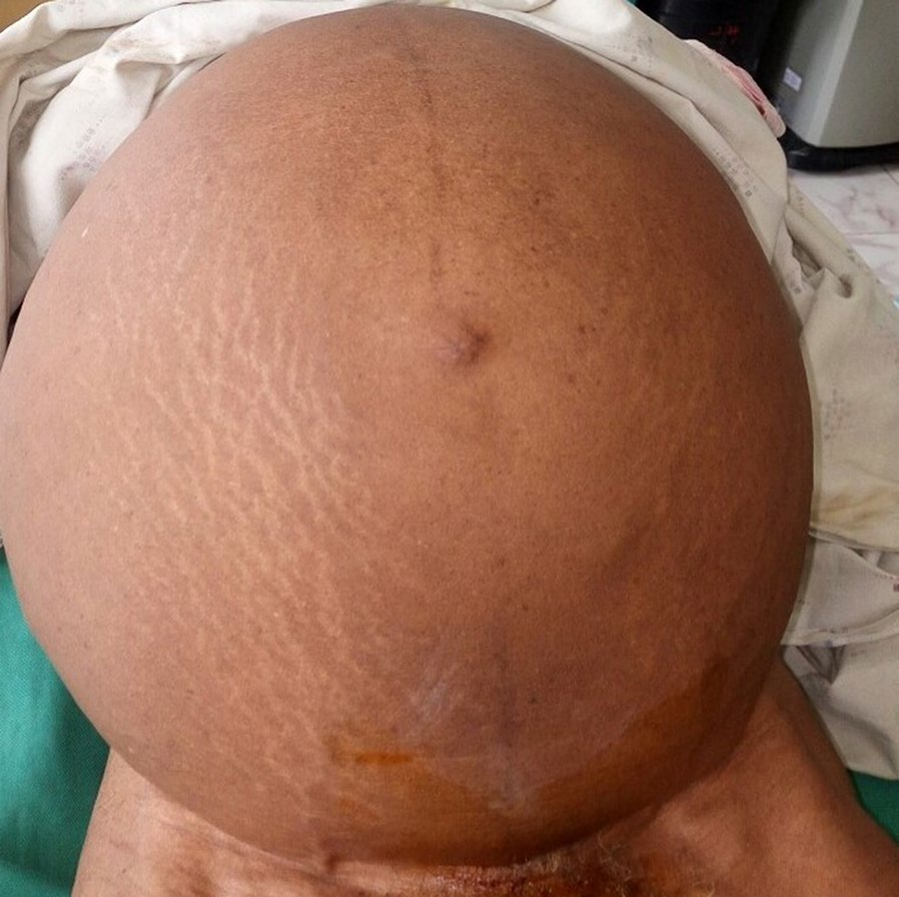
Grossly distended abdomen with full flanks and visible striae

Physical examination revealed pink conjunctivae and anicteric sclerae. Her vital parameters were normal and her weight was 65 kg. Her abdomen was grossly distended with full flanks and visible striae (Fig. 1). It was soft and non-tender with an abdominal girth of 115 cm. Percussion notes were dull over the entire abdomen and fluid thrill was present. Examination of the cardiovascular and urogenital systems were unremarkable.

The following investigations were done: a complete blood count which was normal with a haemoglobin level of 12 g/dl, an abdominal ultrasound scan which was suggestive of a massive fluid-filled multilocular cavity of right ovarian origin with a thin covering and bowel loops shifted against the diaphragm. Other haematological or biochemical serum tests were normal. We concluded on the diagnosis of a right giant multiloculated ovarian cyst.

**Fig. 2:**
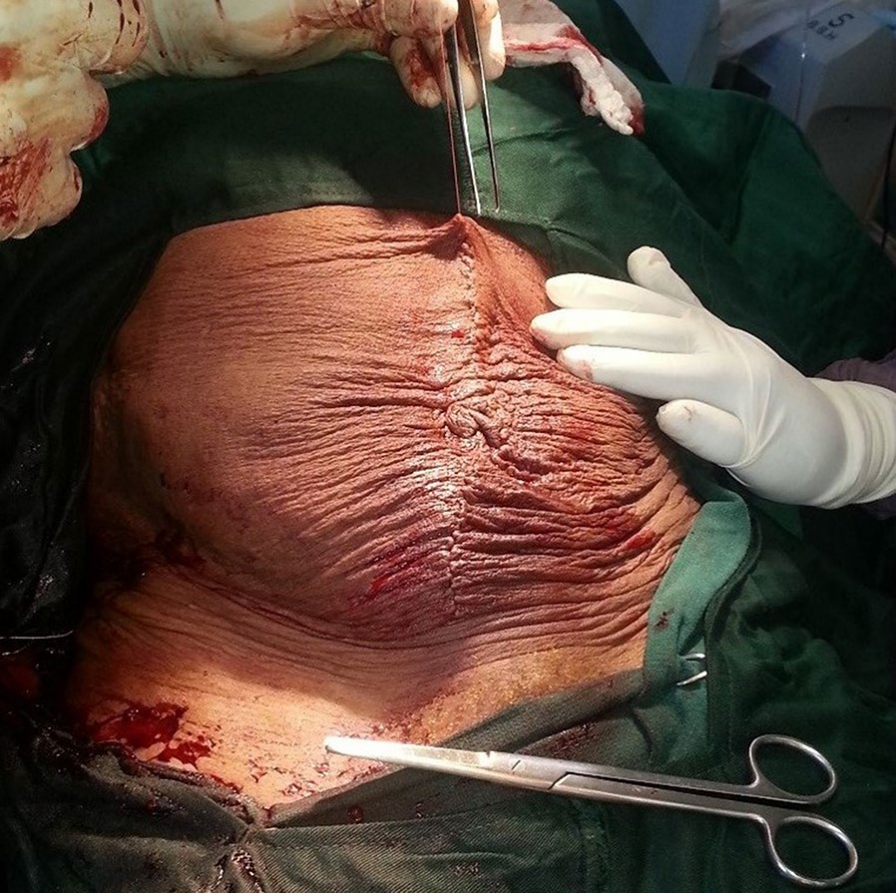
Abdomen showing the midline incision

**Fig. 3:**
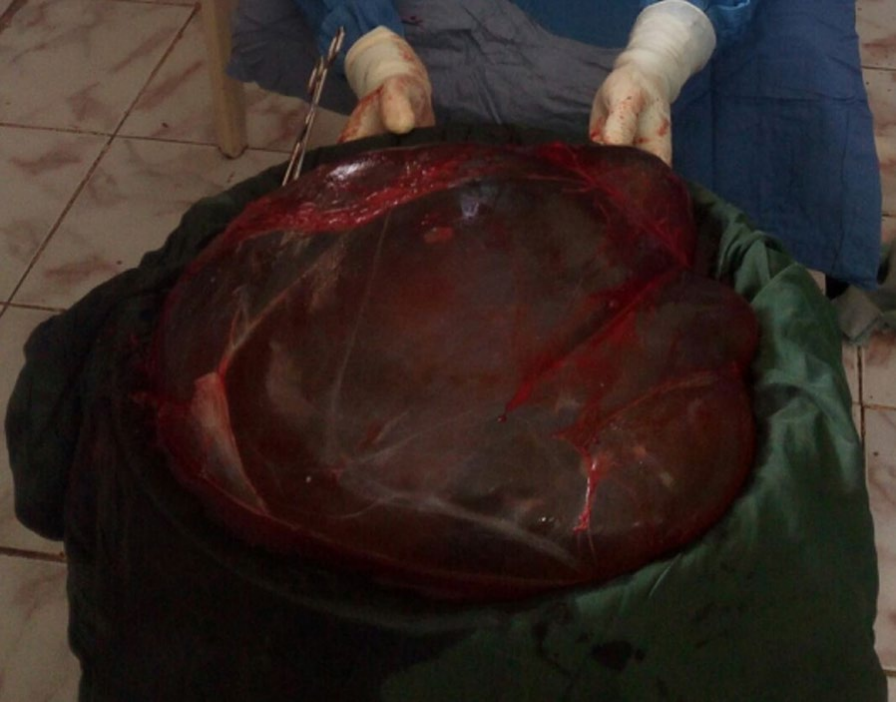
Excised cyst with membranes intact

Following counselling and consent, a right ovarian cystectomy was done through a laparotomy with a midline incision (Fig. 2). Intraoperative findings included a GOC arising from the right ovary with a gelatinous hyper vascularised membrane. The cyst was excised with membranes intact and it measured 55 × 52 × 24 cm and weighed 10.8 kg (Fig. 3). Histopathology revealed mucinous cystadenoma. The postoperative period was uneventful and she was discharged on the 5th postoperative day with a weight of 54 kg.

## Discussion and conclusion

Giant ovarian cysts constitute a challenging condition in general practice because of their nonspecific clinical features and findings from physical examination resulting to a wide range of differential diagnoses [10]. These differential diagnoses include pelvic endometriosis, intra-abdominal pregnancy, intra-abdominal cysts from varying origins (omentum, ovary, kidney, liver, pancreas, cystic lymphangiomas, choledochal cysts), hydronephrosis and accentuated obesity [5, 10]. Despite being asymptomatic, GOCs can cause serious complications like torsion, suppuration, obstruction, and perforation necessitating urgent admission [11]. Many GOCs can present with signs and symptoms of ascites due to their large nature and they are commonly mistaken for it [2–5, 12]. Rosato et al, in their indexed case, showed that giant ovarian neoplasms presenting with very large cystic masses can mimic ascites [3]. In this case, a GOC was equally mistaken for a massive ascites and the diagnosis of an ovarian cyst was retained only after ultrasonographic findings.

Aspiration of the abdominal fluid is used to evaluate its physico-chemical characteristics, cellular composition and the presence of malignant cells and also to relieve abdominal distension and compression of intra-abdominal organs [3]. These aspirations are contra-indicated in the context where intra-abdominal cysts are suspected. Aspiration of an abdominal cyst should be avoided as this could cause complications like infections, bleeding, rupturing of the cyst, increased peritoneal adhesion, or possible dissemination of malignant cells thus making surgical cystectomy more challenging [3–5]. We therefore avoided aspiration of the fluid to relieve the distension or for diagnosis in this case. Pre-operative or intraoperative drainage of GOCs have also proven unsafe and should be avoided. Einenkel et al. [13] laid emphasis on that based on their critical evaluation of many medical cases and their complications.

Radiological imaging studies play a central role in the diagnosis of GOCs. The widespread use of imaging modalities in recent days have resulted in the rarity of GOCs as they are frequently diagnosed and excised earlier. Ultrasound scans (USS) are broadly accepted as diagnostic tools in SSA because they are readily available in many centres and do not pose a challenge in usage [3, 7]. In this case report, the diagnosis of GOC was made through an USS since we lacked advanced imaging modalities like magnetic imaging resonance or computed tomographic scans. USS can also mislead diagnosis necessitating the use of these advanced imaging modalities [3, 4]. The wrong results from USS are likely due to lack of expert radiologists or inexperienced sonographers. According to Elhassan et al. using USS alone requires cautious interpretation in patients in whom there is no clear diagnosis of ascites based on history and examination as it is often operator dependent [4]. Although magnetic imaging resonance and computed tomographic scans are more reliable investigations because they help to evaluate the precise origin of intra-abdominal tumours, to measure the tumour size and relationship with other intra-abdominal structures and to exclude many differential diagnoses [3], they are not readily affordable and are not found in many centres in SSA.

Malignant ovarian cysts constitute over 10% of all GOCs [5] and their signs and symptoms are vague and non-specific especially in early stages of the disease, making it necessary to exclude its possibility in all cases of ovarian cysts. Tumour markers like cancer antigen 125, carcinoembryonic antigens, beta human chorionic gonadotropin and alpha fetoprotein play an important role in early diagnosis, management and follow-up of patients with MOCs [14, 15]. These tumour markers are not frequently included amongst investigations for ovarian cysts in most centres in SSA, although their role in early diagnosis and management of MOCs cannot be overlooked. Though they are not wholly specific or sensitive to ovarian malignancies, they help in determining the various sub-types of ovarian cancers [14, 15]. Since the specificity of each type of tumour marker is typical for the sub-type of ovarian cancer, it becomes important to use more than one tumour marker in determining the actual diagnostic and prognostic status of patients with ovarian neoplasms thus making the use of biomarkers costly [14]. We therefore emphasize on the role of immunohistochemistry in conjunction with clinical and morphologic findings, as it remains paramount in the diagnosis of MOCs.

Occasionally, ovarian cysts reach enormous dimensions without causing any marked symptoms [12]. GOCs as large as 148.6 kg have been reported [2, 16]. With good imaging modalities in recent years, such volumes of ovarian cysts are hardly encountered due to early diagnoses and management [2]. GOCs still occur partly due to late presentation at the hospital and late surgical interventions. Patients present late because debilitating symptoms of GOCs also occur very late. Socio-economic status of most patients in SSA contributes to late presentation and diagnosis. The cultural believes of some patients and the fear of surgical procedures cause many patients to refuse surgery and only accept it when symptoms are totally unbearable [17]. Our patient assumed the increasing abdominal gait was due to obesity which was not a call for concern and she sought medical attention after it persisted for years. Lack of diagnostic facilities in primary settings in SSA also contributes to late diagnosis [10]. Although US scans are relatively common in recent practice, it is apparently not used in many centres in SSA partly because of the lack of the expertise to manage them or their absence in these centres.

This case report emphasizes that large cystic ovarian tumours could present masquerading as an ascites warranting the need to exclude them in all women presenting with massive ascites. In limited resource settings, an abdominopelvic ultrasound scan may assist in the diagnosis. Paracentesis should generally be avoided in cases of ascites where an intra-abdominal cyst is suspected.

## Abbreviations

GOC: giant ovarian cyst
SSA: sub-Saharan Africa
MOC: malignant ovarian cyst
USS: ultrasound scans
